# Relationship between depressive symptoms and anemia among the middle-aged and elderly: a cohort study over 4-year period

**DOI:** 10.1186/s12888-023-05047-6

**Published:** 2023-08-08

**Authors:** Congqi Liu, Ruihao Zhou, Xilin Peng, Tao Zhu, Wei Wei, Xuechao Hao

**Affiliations:** 1 Department of Anesthesiology, National Clinical Research Center for Geriatrics, West China Hospital, Sichuan University, No. 37 Guoxue Xiang, Wuhou District Chengdu, Chengdu, China; 2 The Research Units of West China (2018RU012)-Chinese Academy of Medical Sciences, West China Hospital, Sichuan University, No. 37 Guoxue Xiang, Wuhou District Chengdu, Chengdu, China

**Keywords:** Anemia, Depressive symptoms, CHARLS, Hemoglobin

## Abstract

**Background:**

The association between anemia and depression has been demonstrated in previous studies, but it's still unclear whether depressive symptoms as a hazard factor for anemia. The findings of a large-scale cross-sectional and longitudinal examination of such an association of among the middle-aged and elderly individuals in China were presented in our study.

**Methods:**

The data from China Health and Retirement Longitudinal Study in 2011 and 2015 were evaluated. 10,179 and 5,887 participants were included in cross-sectional and longitudinal study, respectively. According to the World Health Organization, hemoglobin concentrations below 13 g/dL for males and 12 g/dL for females are considered anemia. The research population was separated into two groups based on scores of the 10-item short form of the Center for Epidemiologic Studies Depression Scale (CES-D-10): the group with depressed symptoms had a score of more than 10 points, and the group with depressive disorder had a score of more than 20 points. Multilevel logistic regression analyses were conducted to explore the relationship between anemia and varying degrees of depressive symptoms, utilizing three models based on adjusting for different types of covariates.

**Results:**

In our cross-sectional investigation, depression disorders were more likely to link to the occurrence of anemia (OR, 1.34; 95% CI, 1.02–1.77; *P* = 0.035). Additionally, there seems a linear connection between depression questionnaire scores and hemoglobin concentrations (*r* = - 0.053, *P* < 0.001). Depressive symptom was significantly associated with anemia over 4 years of follow-up, and the more intense the depressive symptoms, the greater the danger of anemia (depressive symptoms group: OR, 1.27; 95% CI, 1.02–1.57, *P* = 0.032; depressive disorder group: OR, 1.59; 95% CI, 1.12–2.25, *P* = 0.010).

**Conclusions:**

Our findings suggest that depression symptoms seem related to anemia in the middle-aged and elderly in China cross-sectionally and longitudinally, and that the risk of anemia increases with the severity of depressive symptoms.

**Supplementary Information:**

The online version contains supplementary material available at 10.1186/s12888-023-05047-6.

## Introduction

In the context of the remarkable growth of global aging, China has the features of a large elderly population and accelerated aging. Inevitably, China will soon encounter strain on the nation's healthcare system brought on by age-related illnesses [1].

Anemia is a condition when the number of red blood cells in human peripheral blood or the hemoglobin concentration is below the lower limit of normality. Anemia is a global public health issue of grave importance. In the 2013 survey, 27% of the world's total population was anemic, with the majority residing in underdeveloped countries [2]. Hemoglobin is necessary for maintaining tissue oxygen metabolism. Thus, weariness, shortness of breath, palpitations, and arrhythmias are the most prevalent clinical symptoms of anemia [3]. Anemia is often associated with chronic diseases and malnutrition conditions such as cancer, renal failure, infection, and neurological illnesses [4]. Fatigue and weakness induced by anemia have a detrimental effect on life quality and the capacity to complete daily tasks. Relevant research has demonstrated that anemia is linked to worsened clinical outcomes, quality of life even increased mortality [5].

Depression is a severe psychological disorder characterized by symptoms such as low mood, loss of energy, low self-esteem, as well as physical and psychological sluggishness. Global Burden of Disease Study 2019 (GBD 2019) revealed that depressive disorder was one of the most disabling mental illnesses [6]. The health condition and quality of life of elderly individuals are obviously impacted by depression and its associated physical discomfort and psychological disorders [7]. Because of its high incidence and serious clinical outcome, depressed people must be a priority category for the public health system, particularly the senior depressed population. Relevant research has demonstrated that persons who are depressed frequently have a poor appetite and malnutrition, which can cause can cause nutritional deficits that can result in a lack of iron intake, potentially leading to the occurrence of anemia [8]. Moreover, depressed individuals are more likely to engage in harmful health-related behaviors and lifestyles, such as smoking, excessive alcohol consumption, and reduced sleep duration. The ensuing diseases (such as chronic pain, cardiovascular disease, liver disease, sleep disturbance etc.) may contribute to the development of anemia [9–11]. According to a pertinent research report, the cytokine known as leukemia inhibitory factor 6 (IL-6) participates in the transmission of brain signals associated with “maladaptive behavior” and exhibits a broad correlation with depressive symptoms [12]. Findings from a Mendelian randomization study indicate a link between the presence of depression and heightened levels of inflammatory responses [13]. Notably, elevated levels of inflammation are among the factors contributing to anemia [14].

Previous cross-sectional research has proven the positive relationship between depression and anemia [15, 16] while a study concluded that anemia was not significantly related to depression [17]. Further study into this relationship is still required because we found that previous observational studies with participants concentrated on specialized populations such as healthy adults, menopausal women, pregnant women, the very elderly or individuals with specific diseases [16, 18–21].

In sum, this research intends to investigate whether depressive symptoms are related to anemia. Furthermore, in order to explore the relationship between anemia and the different degree of depressive symptoms, participants in our study were separated into different groups. We also hypothesized that in cross-sectional studies, more severe depression was more associated with anemia and that depression levels were linearly related to hemoglobin concentrations. Additionally, we assumed that depressive symptoms would increase the risk of anemia after 4 years of follow-up. To further demonstrate the association between depressive symptoms and anemia by presenting more persuasive evidence, a substantial representative sample, China Health and Retirement Longitudinal Study (CHARLS), was utilized in our study [22].

## Methods

### Study sample

Our study was conducted based on data from the CHARLS which is a nationwide representative longitudinal cohort study with individuals aged 45 years or older. The baseline survey (Wave 1) and the third follow-up investigation (Wave 3) were carried out in 2011–2012 and 2015, respectively. Computer-assisted personal interview (CAPI) was used to collect personal and familial information. The cohort included 17,708 respondents in the baseline survey and 14,574 among them accepted return visits in 2015, with a response rate of more than 82%. The Peking University Biomedical Ethics Review Committee (IRB00001052-11015) accepted the study design and methodology and all subjects provided informed consent. More information is available on the CHARLS project website [23].

Basic information, data from health status assessments (including assessments of the severity of depressive symptoms), physical examinations, and blood tests were collected in 2011–2012 (Wave 1) and 2015 (Wave 3). A total of 10,179 eligible individuals were recruited in the cohort study for cross-sectional analysis after excluding those with missing data for gender (*N* = 12) and the scores of depression level (*N* = 2,434), as well as those absent from the hemoglobin test (*N* = 6,175). People whom missing age data or were younger than 45 years were also excluded. Out of the 10,179 participants in the baseline survey, 1,182 dropped out from Wave 3, 2,316 missed the hemoglobin concentration test and 688 were diagnosed with anemia in 2011 (Wave 1). Our final analytic sample finally included 5,887 participants followed up in 2015 (Wave 3).

### Blood measurement and definition of anemia

CHARLS collected blood samples from all eligible participants at survey intervals. Therefore, the hematology data analyzed in this analysis were obtained from the 2011–2012 baseline survey and the 2015 follow-up survey. Medically trained workers collected blood samples in centralized facilities (urban locations for disease control at the district level, county centers for disease control, or town/village clinics in rural areas). For most fasting blood specimens, their Complete blood count (CBC) is performed. The mean corpuscular volume (MCV, fL) and hemoglobin concentrations (g/dL) were evaluated using automated analyzers that were accessible at centrally controlled sites. The blood sample were centrifuged to separate plasma and buffy coat and then frozen at -20 °C. Within two weeks, the samples were shipped to the Chinese Center for Disease Control and Prevention in Beijing, where they were stored in a deep freezer at -80 °C. The blood samples were analyzed to determine the presence of various blood biochemical markers, including serum creatinine, glucose, blood lipid, C-reactive protein (CRP), and others. Capital Medical University's Youanmen Center for Clinical Laboratory completed the detection procedures.

The World Health Organization (WHO) defines anemia as a hemoglobin concentration of less than 13 g/dL for men and less than 12 g/dL for women [24].

### Assessment of severity of depressive symptoms

In the baseline survey of CHARLS in 2011, depressive symptoms were assessed using the 10-item short form of the Center for Epidemiologic Studies Depression Scale (CES-D-10). The CES-D-10 is a frequently used self-report tool for assessing depressed symptoms in epidemiological studies with large sample sizes [25]. It has been found to have strong reliability and validity among China's older population, as demonstrated in articles and journals [26]. Participants were requested to indicate "how frequently you had this sensation during the last week" on a scale of 0 to 3 points for each item, ranging from 0 to 30 points. The questions “I feel hopeful about the future” and “I was happy” were reverse-scored [22]. The ten questions were grouped into three types of depression symptoms: physical symptom, depressive emotion, and optimistic mood. The ten questions were summarized into three types of depression symptoms: physical symptoms, depressive emotion, and optimistic mood. A higher score indicates more severe depressive symptoms within each symptom type. Based on the total score of the respondents' responses to CES-D-10, they were divided into the depressive disorder (DD) group (equal to or greater than 20 scores), depressive symptom (DS) group (equal to or greater than 10 points), and non-depressive symptom (NDS) group (less than 10 points) [27].

### Other covariates

Relevant confounders, including demographic, behavioral characteristics, as well as disease-related factors, were collected in Wave 1. The primary factors included age, gender, education level, marital status, smoking status, alcohol consumption, social activity engagement, and co-morbidities. Besides, residence variable includes rural and urban areas. The respondents’ daily sleep duration was divided into four groups: less than 4 h, 4- 6 h, 6 -8 h, and more than 8 h. Participation of social activities in last month was also recorded. Body mass index (BMI) was calculated using the standard formula: weight in kilograms divided by height in meters squared (kg/m^2^). BMI was categorized into four categories of “obesity”, “overweight”, “regular weight” and “underweight” according to the standard of WHO. Hypertension was diagnosed if systolic blood pressure was equal to or greater than 140 mmHg or diastolic blood pressure was equal to or greater than 90 mmHg for three consecutive measurements during physical examination, or self-reported history and history of taking the antihypertensive drug. The criteria for diabetes were a self-reported history of diagnosis, use of injectable insulin or antidiabetic medications, fasting plasma glucose levels below 126 mg/dL, or non-fasting plasma glucose levels above 200 mg/dL. When the abdominal circumference of 90 cm or more for males or 80 cm or more for females was considered abdominal obesity. Dyslipidemia of at least 200 mg/dl, cholesterol of at least 240 mg/dL, high-density lipoprotein of at least 40 mg/dL, or low-density lipoprotein of at least 160 mg/dL were all considered to have dyslipidemia. Chronic kidney disease (CKD) was defined as a history of diagnosis or an eGFR (estimated by the Cockcroft-Gault equation) lower than 90 ml/min. Chronic pain is defined as the experience of moderate or severe pain persisting for an extended period of time. Given the susceptibility of hemoglobin to various diseases, we collected other comorbidities' history based on self-reported diagnosis and treatment history, including heart disease, chronic liver disease, chronic lung disease, stroke, asthma, etc. There are 14 different types of chronic illnesses in total.

### Statistical analysis

Comparisons among different groups were conducted by χ2-test or Wilcoxon rank sums tests for categorical variables. For continuous variables, Student’s t-test, analysis of variance, or the Kruskal–Wallis test was used. We utilized multilevel logistic regression analysis to investigate the relationship between anemia and different degrees of depressive symptoms. The model with adjusted demographic variables was designated as Model 1. Model 2 was adjusted for demographics and behavioral variables and Model 3 was adjusted for demographics, life behavior, and disease-related variables. The odds ratio (OR) and 95% confidence intervals (CI) were computed, and a *P*-value of 0.05 was considered statistically significant.

Propensity Score Matching (PSM) is a method of sensitivity analysis method, which is capable of completely balancing the distribution of variables across multiple target populations [28]. The likelihood of the sample being the intervention group is represented by the propensity score. The logit model was used to determine it until the observable variable X is available. The calculation equation is as follows:

$$\mathrm{P }(\mathrm{Xi}) =\mathrm{ Pr }(\mathrm{ Di }=1 |\mathrm{ Xi})=\mathrm{ e\beta xi}1 +\mathrm{ e\beta xi}$$

X represents the matrix of control variables. D is the indicator variable, where D = 1 represents the intervention group and D = 0 represents the control group (in this study, D = 1 represents depressive symptoms and D = 0 represents no depression). Kernel matching, nearest neighbor matching, and radius matching are typical matching approaches. To test the robustness of the results, our study adopted the nearest neighbor matching (1:2 and 1:3), caliper range set to 0.05.

Besides, in order to closely investigate the relationship and evaluate any potential disparities between different gender and different age groups, subgroup analyses were conducted.

All statistical analysis was performed by Stata (version 17.0; Stata Corp, College Station, TX, USA).

## Results

### General description

**Fig. 1:**
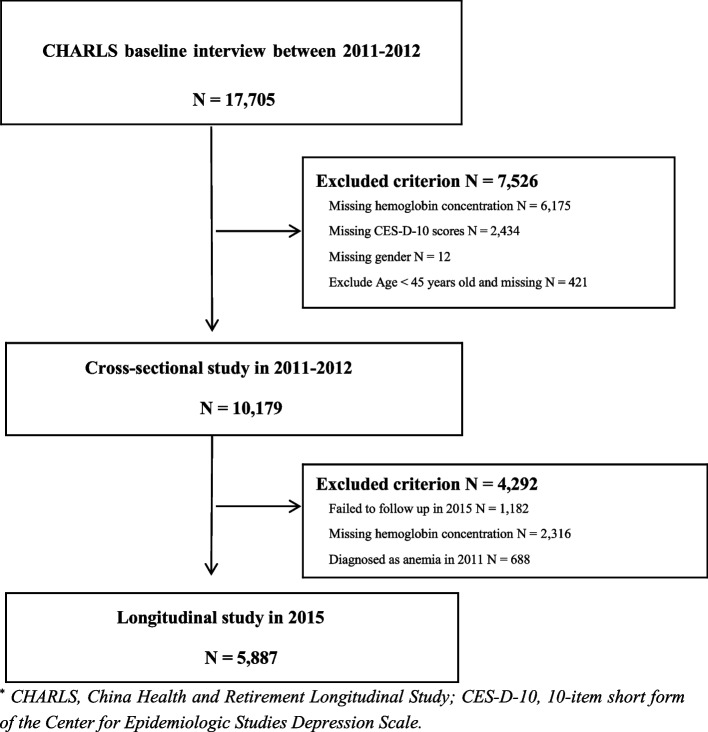
Flow chart of participants through the study

Our cross-sectional research comprised 10,179 participants, including 4,794 males and 5,385 females with a mean age of 59.28 ± 9.37 years. The details of the research sample selection procedure are illustrated in Fig. 1.

NDS, DS, and DD accounted for 61.81 percent (6,292), 30.75 percent (3,130), and 7.44 percent (757) of the total population, respectively. NDS, DS, and DD accounted for 61.81 percent (6,292), 30.75 percent (3,130), and 7.44 percent (757) of the total population, respectively. Moreover, the scores for somatic symptoms, depressive mood, and positive emotion were significantly higher in the DS and DD groups compared to the NDS group. In the DS and DD groups, the mean hemoglobin concentrations were 14.26 ± 2.23 g/dL and 14.21 ± 2.30 g/dL, respectively, which were notably lower than the NDS group’s mean concentration of 14.49 ± 2.14 g/dL.

According to our findings, anemia and depressive symptoms are more prevalent among older, less educated, rural residents who are more likely to be female. In addition, the anemia group differed significantly from the non-anemia group in terms of smoking status, and alcohol consumption, social engagements, nutritional status, CRP, MCV as well as chronic disease including hypertension, dyslipidemia, CKD and chronic pain (Table 1, Supplement Table 1).

**Table 1:** Baseline characteristics of participants by depressive symptom status (2011, *N* = 10,179)

Variables	Assignment description		Depressive Symptoms			
		Total *N* = 10,179	^*^NDS group *N* = 6292 (61.81%)	^*^DS group *N* = 3130 (30.75%)	^*^DD group *N* = 757 (7.44%)	*P*-value
Anemia, %(n)		12.54(1,276)	11.59(729)	13.93(436)	14.66(111)	< 0.001
Hemoglobin (g/dL), mean (^*^SD)		14.40(2.18)	14.49(2.14)	14.26(2.23)	14.21(2.30)	< 0.001
Age, year, mean (SD)		59.05(10.15)	58.64(9.24)	60.12(9.48)	61.11(9.41)	< 0.001
Age, year, %(n)	45—60	55.08(5,607)	58.06(3,653)	50.96(1,595)	47.42(359)	< 0.001
	≥ 60	44.92(4,572)	41.94(2,639)	49.04(1,535)	52.58(398)	
Gender, %(n)	Male	47.10(4,794)	52.38(3,296)	39.87(1,248)	33.03(250)	< 0.001
	Female	52.90(5,385)	47.62(2,996)	60.13(1,882)	66.97(507)	
Educational level, %(n)	Illiterate	27.73(2,822)	23.33(1,468)	32.82(1,027)	43.20(327)	< 0.001
	Primary education	41.08(4,181)	39.31(2,473)	44.84(1,403)	40.29(305)	
	Secondary education	29.52(3,004)	35.11(2,209)	21.44(671)	16.38(124)	
	Higher education	1.65(168)	2.21(139)	0.89(28)	0.13(1)	
	Postgraduate education	0.02(2)	0.03(2)	0.00 (0)	0.00(0)	
Marital status, %(n)	Single	0.72(73)	0.57(36)	0.96(30)	0.92(7)	< 0.001
	Married	88.80(9,039)	91.26(5,742)	86.13(2,696)	79.39(601)	
	Divorced	0.63(64)	0.49(31)	0.73(23)	1.32(10)	
	Widowed	9.85(1,003)	7.68(483)	12.17(381)	18.36(139)	
Residence, %(n)	Rural	80.90(5,032)	77.34(2,914)	84.83(1,649)	92.32(469)	< 0.001
	Urban	19.10(1,140)	22.66(3,378)	15.17(1,481)	7.68(288)	
Smoking status, %(n)	Never	60.25(6,133)	57.84(3,639)	63.29(1,981)	67.77(513)	< 0.001
	Quit	9.23(940)	9.55(601)	8.88(278)	8.06(61)	
	Current	30.52(3,106)	32.61(2,052)	27.83(871)	24.17(183)	
Alcohol consumption, %(n)	Never	67.12 (6,832)	63.72(4,009)	71.88(2,250)	75.69(573)	< 0.001
	Less than once a month	7.78 (792)	8.33(524)	6.96(218)	6.61(50)	
	More than once a month	25.10 (2,555)	27.96(1,759)	21.15(662)	17.70(134)	
Social activities engagement, %(n)	Yes	50.86(5,177)	54.39(3,422)	46.52(1,456)	39.50(299)	< 0.001
	No	49.14(5,002)	45.61(2,870)	53.48(1,674)	60.50(458)	
Sleep duration at night, hours, %(n)	0 ~ 4	7.85(795)	3.78(237)	12.62(392)	22.19(166)	< 0.001
	4 ~ 6	21.96(2,222)	16.67(1,045)	29.39(913)	35.29(264)	
	6 ~ 8	40.37(4,086)	45.28(2,838)	34.39(1,068)	24.06(180)	
	≥ 8	29.82(3,018)	34.26(2,147)	23.60(733)	18.45(138)	
^*^BMI degree, %(n)	Underweight	5.85 (595)	4.66(293)	7.51(235)	8.85(67)	< 0.001
	Normal weight	35.44 (3,607)	34.15(2,149)	38.18(1,195)	34.74(263)	
	Overweight	18.73 (1,907)	19.50(1,227)	17.60(551)	17.04(129)	
	Obesity	39.98 (4,070)	41.69(2623)	36.71(1,149)	39.37(298)	
Co-morbidities, %(n)	Yes	57.48(5,851)	49.09(3,089)	68.98(2,159)	79.66(603)	< 0.001
	No	42.52(4,328)	50.91(3,203)	31.02(971)	20.34(154)	
Hypertension, %(n)		46.71(4,755)	46.07(2,899)	47.60(1,490)	48.35(366)	0.241
Abdominal adiposity, %(n)		45.13(4,594)	44.88(2,824)	45.30(1,418)	46.50(352)	0.681
Diabetes, %(n)		15.17(1,544)	14.51(913)	15.85(496)	17.83(135)	0.025
Dyslipidemia, %(n)		43.12(4,389)	43.93(2,764)	41.34(1,294)	43.73(331)	0.054
^*^CKD, %(n)		57.94(5898)	56.09(3529)	60.99(1909)	60.77(460)	< 0.001
Cancer, %(n)		0.98(100)	0.83(52)	1.18(37)	1.45(11)	0.101
Chronic pain, %(n)		25.97(2,643)	13.81(869)	40.00(1,252)	68.96(522)	< 0.001
^*^CRP (mg/L), mean (SD)		2.77(7.46)	2.69(7.26)	2.90(7.77)	2.82(6.70)	0.513
^*^MCV, mean (SD)		90.58(8.55)	90.75(8.31)	90.31(8.91)	90.66(9.60)	0.032
^*^CES-D-10 scores, mean (SD)		8.61(6.41)	4.43(2.78)	13.57(2.75)	22.87(2.56)	< 0.001
Physical symptoms scores		4.46(3.68)	2.23(1.97)	7.15(2.22)	11.85(1.86)	< 0.001
Depressive emotion scores		1.90(2.14)	0.72(1.02)	3.21(1.66)	6.31(1.78)	< 0.001
Positive mood scores		2.25(1.96)	1.48(1.65)	3.20(1.70)	4.71(1.43)	< 0.001

^*^Abbreviation: *NDS* non-depressive symptom, *DS* depressive symptom, *DD* depressive disorder, *BMI* body mass index, *CKD* Chronic kidney disease, *CRP* C-reactive protein, *MCV* Mean Corpuscular Volume, *CES-D-10* 10-item short form of the Center for Epidemiologic Studies Depression Scale

^*^P-value less than 0.05 was defined as significant

^*^Variables are presented as percentages (number), or mean (SD, standard deviation)

The overall mean score of CES-D-10 was 8.61 ± 6.41, the somatic symptoms was 4.46 ± 3.68, the depression mood was 1.90 ± 2.14 and positive emotion was 2.25 ± 1.96. The mean score of CES-D-10 in the anemia group (9.2 1 ± 6.55) was obviously higher than the non-anemia group (8.53 ± 6.39). Besides, the scores of somatic symptoms, depressed mood, and positive emotion in the anemia group were significantly higher than those in the non-anemia group (Supplement Table 2).

### Cross-sectional relationship between different degrees of depressive symptoms and anemia (Wave 1, 2011)

**Fig. 2:**
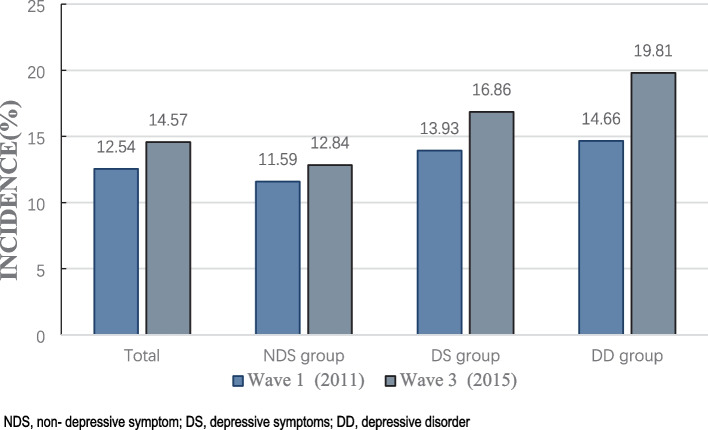
Incidence of anemia among participants in cross-sectional study (2011) and longitudinal study (2015)

In the cross-sectional study, persons in the DD group or DS group had much lower hemoglobin concentration than individuals in the NDS group, and the prevalence of anemia in the NDS group, DS group, and DD group was 11.59% (729), 13.93% (436), and 14.66% (111) respectively (*p*-value < 0.001, Fig. 2). In addition, the anemia group scored higher than the without anemia group on each CES-D-10 item (Supplement Table 1).

Adjusting for confounding variables, Table 2 displays the results of a multilevel logistic regression study investigating the connection between different degrees of depression and anemia. Our findings indicate that experiencing a depressive disorder is a risk factor for anemia. After adjusting for demographic variables (Model 1), participants in the DD group had higher rates of anemia compared to those in the NDS group (OR, 1.39; 95% CI, 1.07–1.80; *P* = 0.006). These results remained consistent, and the relationship between anemia and the DD group remained significant even after adjusting for demographic and behavioral variables (Model 2) (OR, 1.36; 95% CI, 1.04–1.78; *P* = 0.025), as well as demographic, behavioral, and disease-related variables (Model 3) (OR, 1.34; 95% CI, 1.02–1.77; *P* = 0.035). The multilevel regression analyses also indicated that higher CES-D-10 scores, somatic symptoms, depressed mood, and positive emotion scores were substantially linked to a higher risk of anemia in Model 1 and Model 2, but statistically insignificant in Model 3.

**Table 2:** The relationship between different depressive symptoms group, scores and anemia in cross-sectional study (2011)

	Model 1^a^		Model 2^b^		Model 3^c^	
	^*^OR (95% ^*^CI)	*P*	OR (95% CI)	*P*	OR (95% CI)	*P*
^*^NDS group (*N* = 6,292)	1(reference)		1(reference)		1(reference)	
^*^DS group (*N* = 3,130)	1.07(0.91–1.27)	0.411	1.09(0.91–1.29)	0.346	1.06(0.89–1.27)	0.492
^*^DD group (*N* = 757)	1.39(1.07–1.80)	0.006	1.36(1.04–1.78)	0.025	1.34(1.02–1.77)	0.035
^*^CES-D-10 scores	1.01(1.00–1.02)	0.038	1.01(1.00–1.02)	0.048	1.01(0.99–1.02)	0.356
Physical symptoms scores	1.02(1.01–1.04)	0.008	1.02(1.00–1.04)	0.025	1.00(0.98–1.03)	0.119
Depressive emotion scores	1.04(1.00–1.07)	0.039	1.04(1.00–1.07)	0.043	1.03(1.00–1.07)	0.076
Positive mood scores	1.04(1.01–1.07)	0.010	1.03(1.00–1.06)	0.046	1.02(0.99–1.05)	0.156

^*^Abbreviation: *OR* odds ratio, *CI* confidence intervals, *NDS* non-depressive symptom, *DS* depressive symptom, *DD* depressive disorder, *CES-D-10* Center for Epidemiologic Studies Depression Scale

^a^Adjusted for demographic variables (including age, gender, education, marital status, residence)

^b^Adjusted for demographic and behavioral variables (including smoking status, alcohol consumption, social participation and daily sleep duration)

^c^Adjusted for demographic, behavioral and disease-related variables (including BMI, CRP, hypertension, diabetes, dyslipidemia, abdominal obesity, chronic lung disease, heart disease, stroke, cancer, chronic kidney disease, hepatopathy, asthma and chronic pain)

**Fig. 3:**
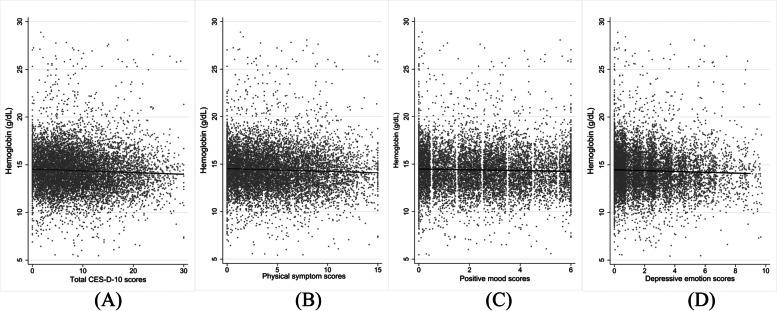
The linear connection between depressive symptoms scores and hemoglobin concentration in cross-sectional study (2011)

Our research also investigated to determine if the score of depressive symptoms assessment and hemoglobin concentration had a linear connection in cross-sectional study. It revealed a minor but significant linear correlation between hemoglobin concentration and the CES-D-10 total scores (Fig. 3 (A): *r* = - 0.053, *P* < 0.001), somatic symptoms scores (Fig. 3 (B): *r* = - 0.049, *P* < 0.001), depression mood scores (Fig. 3 (C): *r* = - 0.043, *P* < 0.001) as well as in positive emotion scores (Fig. 3 (D): *r* = - 0.038, *P *< 0.001.

### Relationship between different degrees of depressive symptoms and anemia on four years follow-up in 2015 (Wave 3, 2015)

A total of 1,182 people failed to attend the visit, and 2,316 failed to collect hemoglobin samples. Following the exclusion of participants who had been diagnosed with anemia, a cohort of 5887 individuals was included in the longitudinal cohort analysis.

In Wave 3, the percentage of newly diagnosed anemia in the follow-up individuals, the NDS group, the DS group, and the DD group with depression were 14.57% (858), 12.84% (471), 16.86% (302), and 19.81% (85) respectively (Fig. 2). Moreover, we conducted a multinomial logistic regression analysis in 2015 to investigate the relationship between different degrees of depression in longitudinal research. After adjusting for all relevant factors (Model 3), our results showed that both the DS group (OR, 1.27; 95% CI, 1.02–1.57; *P* = 0.032) and the DD group (OR, 1.59; 95% CI, 1.12–2.25; *P* = 0.010) had significantly higher incidences of anemia compared to the NDS group (Table 3).

**Table3:** Longitudinal association between different depressive symptoms group, scores and anemia (2015)

	Model 1^a^		Model 2^b^		Model 3^c^	
	^*^OR (95% ^*^CI)	*P*	OR (95% CI)	*P*	OR (95% CI)	*P*
^*^NDS group (*N* = 3,667)	1(reference)		1(reference)		1(reference)	
^*^DS group (*N* = 1,791)	1.35(1.10–1.66)	0.004	1.34(1.08–1.65)	0.008	1.27(1.02–1.57)	0.032
^*^DD group (*N* = 429)	1.70(1.22–2.36)	0.002	1.66(1.18–2.34)	0.003	1.59(1.12–2.25)	0.010
^*^CES-D-10 scores	1.03(1.02–1.05)	< 0.001	1.03(1.02–1.04)	< 0.001	1.03(1.01–1.04)	0.001
Physical symptoms scores	1.05(1.03–1.08)	< 0.001	1.05(1.02–1.08)	< 0.001	1.04(1.02–1.07)	0.002
Depressive emotion scores	1.05(1.01–1.08)	0.011	1.04(1.01–1.08)	0.024	1.03(0.99–1.07)	0.113
Positive mood scores	1.12(1.07–1.18)	0.002	1.12(1.07–1.18)	< 0.001	1.11(1.05–1.17)	< 0.001

^*^Abbreviation: *OR* odds ratio, *CI* confidence intervals, *NDS* non-depressive symptom, *DS* depressive symptom, *DD* depressive disorder, *CES-D-10* Center for Epidemiologic Studies Depression Scale

^a^Adjusted for demographic variables (including age, gender, education, marital status, residence)

^b^Adjusted for demographic and behavioral variables (including smoking status, alcohol consumption, social participation and daily sleep duration)

^c^Adjusted for demographic, behavioral and disease-related variables (including BMI, CRP, hypertension, diabetes, dyslipidemia, abdominal obesity, chronic lung disease, heart disease, stroke, cancer, chronic kidney disease, hepatopathy, asthma and chronic pain)

The findings also revealed that a significant increase in the risk of anemia with higher CES-D-10 scores, somatic symptom, depressive emotion, and optimistic mood scores, which was similar to the baseline outcomes (Table 3).

### Sensitivity analysis

After conducting propensity score matching (PSM) for the 1:2 and 1:3 matched designs, the cross-sectional study retained 4,845 and 5,097 individuals respectively for sensitivity analysis. In the longitudinal investigation, there were 2,750 and 2,916 participants included for analysis. The characteristics of participants after performing propensity score matching (PSM) for a 1:2 matched design in the cross-sectional and longitudinal studies have been provided in Supplementary Tables 3 and 4, respectively. The significant covariates observed among different depression severity groups at baseline continued to be significant in the population characteristics of the matched participants, indicating their persistent impact on the study outcomes.

In the DS group, the result of the cross-sectional logistic regression analysis regarding the association between depressive symptoms and anemia remained non-significant. However, the results of the association between depressive symptoms and anemia remained significant in the DD group in the cross-sectional study, as well as in both of these groups in the longitudinal study using multivariate logistic regression. The results of PSM in a 1:3 matched design are similar to the above results (Table 4).

**Table 4:** Sensitivity analyses for the relationship between different groups of depressive symptom and anemia

1:2 ^*^PSM	Cross-sectional study (2011)		Longitudinal study (2015)	
	*N* = 4,845		*N* = 2,750	
	^*^OR (95% ^*^CI)	*P*	OR (95% CI)	*P*
^*^NDS group	1(reference)		1(reference)	
^*^DS group	1.07(0.88–1.29)	0.507	1.34(1.06–1.69)	0.015
^*^DD group	1.35(1.02–1.79)	0.035	1.67(1.16–2.39)	0.005
1:3 PSM	Cross-sectional study (2011)		Longitudinal study (2015)	
	*N* = 5,097		*N* = 2,916	
NDS group	1(reference)		1(reference)	
DS group	1.08(0.90–1.29)	0.183	1.30(1.04–1.63)	0.024
DD group	1.36(1.03–1.79)	0.021	1.62(1.14–2.31)	0.007

^*^Abbreviation: *OR* odds ratio, *CI* confidence intervals, *PSM* Propensity Score Matching, *NDS* non-depressive symptom, *DS* depressive symptom, *DD* depressive disorder

After controlling for all relevant covariates, the gender subgroup analysis revealed that among male participants in the cross-sectional study, the DD group in males had a higher likelihood of developing anemia compared to the NDS group (OR, 1.72; 95% CI, 1.11–2.65; *P* = 0.014). Additionally, both the DS group in males (OR, 1.08; 95% CI, 0.83–1.41), as well as the DS group (OR, 1.03; 95% CI, 0.81–1.30) and the DD group (OR, 1.20; 95% CI, 0.84–1.70) in females, also displayed higher likelihoods of developing anemia compared to the NDS group. However, it is important to note that despite these observations, the p-value did not reach statistical significance.

After adjusting for all relevant factors in the longitudinal study cohort, it was found that both males in the DD group (OR, 2.19; 95% CI, 1.27–3.75; *P* = 0.005) and females in the DD group (OR, 1.54; 95% CI, 1.16–2.06; *P* = 0.003) had a higher risk of anemia compared to the NDS group. The female DS group (OR, 1.42; 95% CI, 0.89–2.25) also demonstrated an increased risk of anemia, but these results were not statistically significant after adjusting for all relevant factors.

In contrast to the subgroup analysis based on gender, the age subgroup analysis, after adjusting for relevant factors, showed negative results for both the DS and DD groups across all age categories in both cross-sectional and longitudinal studies.

## Discussion

In a sizable, nationally representative sample, this study first investigated the potential association between varying degrees of depressive symptoms and anemia. Our findings suggest that depression appears to be independently and positively associated with higher odds of baseline anemia and anemia after 4 years of follow-up. Moreover, this association becomes more pronounced with severe depression. Additionally, we found a negative linear correlation between the CES-D-10 score and hemoglobin concentration.

Consistent with previously published research, our cohort study revealed that individuals with anemia were more likely to engage in unhealthy lifestyle behaviors, such as smoking, excessive drinking, and experiencing sleep deprivation. Furthermore, they were also more likely to have other systemic diseases (Supplement Table 1). Notably, most of these characteristics were more prevalent in both the DS and DD groups compared to the NDS group. Relevant studies have shown that depression may potentially result in physical health problems, including the occurrence of anemia, through interactions with lifestyle behaviors and chronic diseases [29].

Several prior studies supposed that the relationship between depression and anemia is more likely bidirectional rather than one-way [30, 31]. Earlier studies demonstrated that anemia in individuals may contribute to development of depression due to malnutrition. The fact that depression can trigger anemia, though, is frequently overlooked. Our findings support prior studies suggesting a relationship between different degrees of depression and anemia. For example, a population-based study of the elderly by the Italian National Council for Ageing Research (INRCA, Florence, Italy) including 1156 participants aged 65 years and older showed that the risk of anemia rose with the intensity of depressive symptoms after adjusting for confounders [15]. Additionally, a large cross-sectional study of adults without chronic disease and medication also found a significant and strong link between depression and anemia [32]. In the longitudinal cohort study after four years of follow-up, our results show that depressive symptoms are an independent risk factor for anemia.

The research mentioned above did not explore the linear association between hemoglobin concentrations and depression symptom scores. A Chinese study involving 180 veterans showed that hemoglobin concentrations were inversely associated with depression, which was consistent with our findings [18]. This, however, contradicts the findings of Japanese research, whose analysis showed no significant association between hemoglobin concentrations and depressive mood among high-risk older men in need of care [33]. The existence of conflicts may be partially explained by different study samples (such as age, earnings, education degree, etc.), research approach, methods of statistical analysis, and confounding factor adjustment.

Furthermore, sensitivity analyses were conducted by using the PSM approach. Even after applying PSM, our findings revealed that there remained a robust connection between depression and anemia in the longitudinal study. This implies that depression seems to influence anemia regardless of demographic, behavioral, or disease-related variables. By conducting a gender subgroup analysis, we can speculate that the risk of developing depressive symptoms associated with anemia in the female population may not be immediate, but rather have a long-term manifestation.

Malnutrition is recognized as one of the most prevalent anemia danger factors. B vitamins have been identified as key cofactors in the generation and regulation of dopaminergic and serotonergic neurotransmitters [20, 34]. Both neurotransmitters play an important role in mood adjustment as well as clinical depressive and anxiety disorders [35]. A previous study examining the impact of various nutrient deficiencies on depressive symptoms revealed that the combination of anemia with deficiencies in vitamin B6 and/or folate is associated with indicators of depressed mood and negative emotions [36]. Further research is needed to establish whether depressive symptoms can potentially cause anemia by reducing levels of B6 or folate.

Previous research has revealed that the bodies of individuals with depression are in a state of high inflammatory response for an extended period. Meanwhile, chronic inflammation is regarded as one of the most typical causes of anemia [37]. The inflammatory process could impair iron intake, storage, and metabolism by releasing a substantial number of inflammatory cells and pro-inflammatory factors ( particularly IL-6), which limit red blood cell production or decrease their lifespan [20]. There is growing evidence that inflammation plays a significant role in the genesis and progression of stress-related diseases [11, 38]. At the same time, the level of inflammatory response is also closely related to the occurrence of depressive symptoms [13, 39]. Human inflammatory response levels are partly reflected by C-reactive protein [40]. In this study, high C-reactive protein levels were significantly associated with more severe depressive symptoms and anemia.

Based on previous investigations, the induction of depressive symptoms can result in adverse alterations in hemodynamics and autonomic function, including activation of central adrenergic responses, due to acute stress physiological regulation. The bone marrow microenvironment may be impacted by this pathophysiological process, potentially hindering erythropoiesis [41, 42].

This study holds several notable strengths. Firstly, the sample group was representative on a national level, offering diversity and geographic dispersity and encompassing groups of middle-aged and older people with a range of health conditions. Secondly, unlike to earlier research, our study is the first cross-sectional and longitudinal cohort study to investigate the relationship between depressive symptoms and anemia within an Asian community. Additionally, the sample population was divided into groups in this study to examine the relationship between different levels of depression and the prevalence of anemia, ensuring that even those with mild depression were not overlooked. It was further investigated whether the linear connection between hemoglobin concentration and depression scores existed. To enhance the validity of the findings, propensity score matching and subgroup analyses in gender and age analysis were employed. However, there are certain limitations to our study as well. Due to the long distance and time required to transport some blood samples, improper preservation of tubes of venous blood during transit may result in aberrations in blood sample test results. Secondly, we were unable to examine the etiology of anemia because CHARLS did not collect data on blood ferritin, vitamin B12, homocysteine, folic acid, etc. As a consequence, we were unable to categorize anemia in addition to examining the association between depressive symptoms and specific of anemia. Finally, since a significant portion of the data regarding general characteristics, behavior, and disease-related aspects of the sample group were acquired via the questionnaire designed by CHARLS, there is a possibility of participant recall bias influencing the accuracy of the study outcomes.

## Conclusions

In sum, our study suggests a potential association between depressive symptoms and anemia in the middle-aged and elderly in China both cross-sectionally and longitudinally. The findings also indicate that the risk of anemia rises as the severity of depressive symptoms increases. Our research contributes to the early management of depressive mood, which may help prevent the possible detrimental effects of depressive symptoms on the set and progression of anemia. Further investigation are necessary to confirm the causal link between these two conditions and to determine whether treating depression can also improve anemia in the future.

### Supplementary Information

**Additional file 1: Supplement Table 1.** Baseline characteristics of participants by anemia status (2011, *N* = 10,179).

**Additional file 2: Supplement Table 2. **Differences in specific scores of CES-D-10 between anemia and non-anemia group (2011, *N* = 10,179).

**Additional file 3: Supplement Table 3.** Baseline characteristics of participants after PSM for a 1:2 matched design (2011, *N* = 4,845).

**Additional file 4: Supplement Table 4. **Characteristics of participants after PSM for a 1:2 matched design in longitudinal study (2015, *N* = 2,750).

**Additional file 5: Supplement Table 5.** Gender subgroup analysis: the relationship between different depressive symptoms group, scores and anemia in cross-sectional study (2011).

**Additional file 6: Supplement Table 6. **Gender subgroup analysis: longitudinal association between different depressive symptoms groups, scores and anemia (2015).

**Additional file 7: Supplement Table 7. **Age subgroup analysis: the relationship between different depressive symptoms group, scores and anemia in cross-sectional study (2011).

**Additional file 8: Supplement Table 8. **Age subgroup analysis: longitudinal association between different depressive symptoms groups, scores and anemia (2015).

## Data Availability

The datasets supporting the conclusions of this article are publicly available in the https://charls.charlsdata.com/index/zh-cn.html.

## Abbreviations

BMI: Body mass index
CAPI: Computer-assisted personal interview
CBC: Complete blood count
CES-D-10: Center for Epidemiologic Studies Depression Scale
CHARLS: China Health and Retirement Longitudinal Study
CI: Confidence intervals
CKD: Chronic kidney disease
CRP: C-reactive protein
DD: Depressive disorder
DS: Depressive symptom
GBD: Global Burden of Disease
MCV: Mean corpuscular volume
NDS: Non-depressive symptom
OR: Odds ratio
PSM: Propensity score matching
WHO: World Health Organization
