# Supplementary material for: Relationship between depressive symptoms and anemia among the middle-aged and elderly: a cohort study over 4-year period
Source: BMC Psychiatry. 2023 Aug 8;23:572. doi: 10.1186/s12888-023-05047-6 (PMC10408197; doi:10.1186/s12888-023-05047-6)
Supplement: Supplementary file 1 — Additional file 1: Supplement Table 1. Baseline characteristics of participants by anemia status (2011, N = 10,179). [file 12888_2023_5047_MOESM1_ESM.docx]

| **Supplement Table 1 Baseline characteristics of participants by anemia status (2011, N = 10,179)** | | | | | |
| --- | --- | --- | --- | --- | --- |
| Variables | Assignment description | Total N = 10,179 | Non-anemia N=8903 (87.46%) | Anemia  N=1276 (12.54%) | P-value |
| Depression severity | NDS group | 61.81(6,292) | 62.48(5,563) | 57.13(729) | < 0.001 |
|  | DS group | 30.75(3,130) | 30.26(2,694) | 34.17(436) |  |
|  | DD group | 7.44(757) | 7.26(646) | 8.70(111) |  |
| CESD-10 scores, mean (*^*^*SD) |  | 8.61(6.41) | 8.53(6.39) | 9.21(6.55) | < 0.001 |
| Physical symptoms scores |  | 4.46(3.68) | 4.42(3.67) | 4.76(3.75) | 0.002 |
| Depressive emotion scores |  | 1.90(2.14) | 1.88(2.12) | 2.05(2.22) | 0.009 |
| Positive mood scores |  | 2.25(1.96) | 2.23(1.96) | 2.39(1.94) | 0.004 |
| Female, %(n) |  | 52.90(5,385) | 52.03(4,632) | 59.01(753) | < 0.001 |
| Age, year, mean (SD) |  | 59.05(10.15) | 58.96(9.07) | 61.52(10.96) | < 0.001 |
| Age, year, %(n) | 45-59 | 55.08(5,607) | 56.24(5,007) | 47.02(600) | < 0.001 |
|  | ≥60 | 44.92(4,572) | 43.76(3,896) | 52.98(676) |  |
| Educational level, %(n) | Illiterate | 27.73(2,822) | 26.87(2,392) | 33.70(430) | < 0.001 |
|  | Primary education | 41.08(4,181) | 40.84(3,636) | 42.79(546) |  |
|  | Secondary education | 29.52(3,004) | 30.52(2,717) | 22.49(287) |  |
|  | Higher education | 1.65(168) | 1.74(155) | 1.02(13) |  |
|  | Postgraduate education | 0.02(2) | 0.02(2) | 0.00(0) |  |
| Marital status, %(n) | Single | 0.72(73) | 0.71(63) | 0.78(10) | 0.1578 |
|  | Married | 88.80(9,039) | 88.98(7,922) | 87.54(1,117) |  |
|  | Divorced | 0.63(64) | 0.67(60) | 0.31(4) |  |
|  | Widowed | 9.85(1,003) | 9.64(858) | 11.36(145) |  |
| Residence, %(n) | Rural | 80.90(5,032) | 80.47(4,375) | 83.91(657) | 0.022 |
|  | Urban | 19.10(1,188) | 19.53(1,062) | 16.09(126) |  |
| Smoking status, %(n) | Never | 60.25(6,133) | 59.50(5,297) | 65.52(836) | < 0.001 |
|  | Quit | 9.23(940) | 9.24(823) | 9.17(117) |  |
|  | Current | 30.52(3,106) | 31.26(2,783) | 25.31(323) |  |
| Alcohol consumption, %(n) | Never | 67.12(6,832) | 66.47(5,918) | 71.63(914) | < 0.001 |
|  | Less than once a month | 7.78(792) | 7.91(704) | 6.90(88) |  |
|  | More than once a month | 25.10(2,555) | 25.62(2,281) | 21.47(274) |  |
| Social activities engagement, %(n) | yes | 50.86(5,177) | 51.83(4,614) | 44.12(563) | < 0.001 |
|  | no | 49.14(5,002) | 48.17(4,289) | 55.88(713) |  |
| Sleep duration at night , hour, %(n) | 0-4h | 7.85(795) | 7.75(686) | 8.58(109) | 0.009 |
|  | 4-6h | 21.96(2,222) | 21.74(1,924) | 23.45(298) |  |
|  | 6-8h | 40.37(4086) | 40.52(3,586) | 39.34(500) |  |
|  | ≥ 8h | 29.82(3,018) | 29.99(2,654) | 28.64(364) |  |
| *^*^*BMI degree, %(n) | Underweight | 5.85 (595) | 5.23(466) | 10.11(129) | < 0.001 |
|  | Normal weight | 35.44(3,607) | 34.55(3,076) | 41.61(531) |  |
|  | Overweight | 18.73(1,907) | 18.97(1,689) | 17.08(218) |  |
|  | Obesity | 39.98 (4,070) | 41.24(3,672) | 31.19(398) |  |
| Abdominal Adiposity, %(n) |  | 45.13(4,594) | 45.83(4,080) | 40.28(514) | < 0.001 |
| Hypertension, %(n) |  | 46.71(4,755) | 47.59(4,237) | 40.60(518) | < 0.001 |
| Diabetes, %(n) |  | 15.17(1,544) | 15.13(1,347) | 15.44(197) | 0.773 |
| Dyslipidemia, %(n) |  | 43.12(4,389) | 44.15(3,931) | 35.89(458) | < 0.001 |
| *^*^*CKD, %(n) |  | 57.94(5,898) | 57.04(5,078) | 64.26(820) | < 0.001 |
| Cancer, %(n) |  | 0.98(100) | 0.94(84) | 1.25(16) | 0.293 |
| Chronic pain, %(n) |  | 25.97(2,643) | 25.33(2,255) | 30.41(388) | < 0.001 |
| Co-morbidities, %(n) |  | 57.48(5,851) | 57.35(5,106) | 58.39(745) | 0.485 |
| *^*^*CRP (mg/L), mean (SD) |  | 2.77(7.46) | 2.61(6.58) | 3.88(11.85) | < 0.001 |
| *^*^*MCV, mean (SD) |  | 90.58(8.55) | 91.23(7.74) | 86.07(11.89) | < 0.001 |
| Hemoglobin(g/dL), mean (SD) |  | 14.40(2.18) | 14.84(1.91) | 11.30(1.24) | < 0.001 |
| *^*^Variables are presented as percentages (number), or mean (SD).* | | | | | |
| *^*^Abbreviation: BMI, body mass index; CKD, Chronic kidney disease; CRP, C-reactive protein; MCV, Mean Corpuscular Volume; NDS, non-depressive symptom; DS, depressive symptom; DD, depressive disorder; CES-D-10, 10-item short form of the Center for Epidemiologic Studies Depression Scale.* | | | | | |
| *^*^P-value less than 0.05 was defined as significant.* | | | | |  |
