# Supplementary material for: Relationship between depressive symptoms and anemia among the middle-aged and elderly: a cohort study over 4-year period
Source: BMC Psychiatry. 2023 Aug 8;23:572. doi: 10.1186/s12888-023-05047-6 (PMC10408197; doi:10.1186/s12888-023-05047-6)
Supplement: Supplementary file 2 — Additional file 2: Supplement Table 2. Differences in specific scores of CES-D-10 between anemia and non-anemia group (2011, N = 10,179). [file 12888_2023_5047_MOESM2_ESM.docx]

| **Supplement Table2 Differences in specific scores of CES-D-10 between anemia and non-anemia group (2011, N = 10,179)** | | | | |  |
| --- | --- | --- | --- | --- | --- |
| Items | Anemia | Non-anemia | p-value |  |  |
| Total *^*^*CES-D-10 scores | 9.21(6.55) | 8.53(6.39) | < 0.001 |  |  |
| Somatic symptoms | 4.76(3.75) | 4.42(3.67) | 0.002 |  |  |
| I was bothered by things. | 1.10(1.11) | 1.04(1.11) | 0.076 |  |  |
| I had trouble keeping mind. | 1.00(1.11) | 0.94(1.09) | 0.042 |  |  |
| I felt everything I did was an effort. | 1.13(1.18) | 1.02(1.14) | 0.001 |  |  |
| My sleep was restless. | 1.10(1.20) | 1.06(1.20) | 0.283 |  |  |
| I Could not get going. | 0.43(0.85) | 0.36(0.79) | 0.005 |  |  |
| Depressed mood | 2.05(2.22) | 1.88(2.12) | 0.009 |  |  |
| I Felt depressed. | 1.04(1.08) | 0.99(1.08) | 0.088 |  |  |
| I Felt fearful. | 0.39(0.85) | 0.36(0.78) | 0.156 |  |  |
| I Felt lonely. | 0.62(1.00) | 0.54(0.95) | 0.006 |  |  |
| Positive emotion | 2.39(1.94) | 2.23(1.96) | 0.004 |  |  |
| I feel hopeful about the future. | 1.26(1.19) | 1.18(1.20) | 0.017 |  |  |
| I was happy. | 1.13(1.12) | 1.05(1.11) | 0.015 |  |  |
| *^*^Variables are presented as mean (SD).* | | | | | |
| *^*^Abbreviation: CES-D-10, 10-item short form of the Center for Epidemiologic Studies Depression Scale.* | | | |  |  |
| *^*^P-value less than 0.05 was defined as significant.* | |  |  |  |  |
