# Supplementary material for: Relationship between depressive symptoms and anemia among the middle-aged and elderly: a cohort study over 4-year period
Source: BMC Psychiatry. 2023 Aug 8;23:572. doi: 10.1186/s12888-023-05047-6 (PMC10408197; doi:10.1186/s12888-023-05047-6)
Supplement: Supplementary file 3 — Additional file 3: Supplement Table 3. Baseline characteristics of participants after PSM for a 1:2 matched design (2011, N = 4,845). [file 12888_2023_5047_MOESM3_ESM.docx]

| **Supplement Table 3 Baseline characteristics of participants after PSM for a 1:2 matched design (2011, N = 4,845)** | | | | | |
| --- | --- | --- | --- | --- | --- |
| Variables | Assignment description | Depressive Symptoms | | |  |
|  |  | NDS group N=2,411 (49.76%) | DS group N= 1,928 (39.79%) | DD group N=506 (10.45%) | P-value |
| Anemia, %(n) |  | 11.86(286) | 13.49(260) | 17.00(86) | 0.006 |
| Hemoglobin (g/dL), mean (*^*^*SD) |  | 14.37(2.15) | 14.27(2.21) | 14.11(2.52) | 0.022 |
| Age, year, mean (SD) |  | 58.54(9.29) | 60.45(9.99) | 61.35(9.58) | < 0.001 |
| Age, year, %(n) | 45-59 | 58.73(1,416) | 50.47(973) | 47.23(239) | < 0.001 |
|  | ≥60 | 41.27(995) | 49.53(955) | 52.77(267) |  |
| Gender, %(n) | Male | 45.46(1,096) | 42.69(823) | 35.38(179) | < 0.001 |
|  | Female | 54.54(1,315) | 57.31(1,105) | 64.62(327) |  |
| Educational level, %(n) | Illiterate | 23.52(567) | 29.88(576) | 40.51(205) | < 0.001 |
|  | Primary education | 42.68(1,029) | 47.15(909) | 42.09(213) |  |
|  | Secondary education | 32.14(775) | 22.04(425) | 17.19(87) |  |
|  | Higher education | 1.58(38) | 0.93(18) | 0.20(1) |  |
|  | Postgraduate education | 0.08(2) | 0.00(0) | 0.00(0) |  |
| Marital status, %(n) | Single | 0.41(10) | 1.56(30) | 1.38(7) | < 0.001 |
|  | Married | 86.69(2,090) | 77.75(1,499) | 69.17(350) |  |
|  | Divorced | 0.50(12) | 1.14(22) | 1.98(10) |  |
|  | Widowed | 12.40(299) | 19.55(377) | 27.47(139) |  |
| Residence, %(n) | Rural | 83.28(2,008) | 84.85(1,636) | 92.29(467) | < 0.001 |
|  | Urban | 16.72(403) | 15.15(292) | 7.71(39) |  |
| Smoking status, %(n) | Never | 60.60(1,461) | 61.26(1,181) | 65.61(332) | < 0.001 |
|  | Quit | 7.34(177) | 9.13(176) | 9.09(46) |  |
|  | Current | 32.06(773) | 29.62(571) | 25.30(128) | 0.010 |
| Alcohol consumption, %(n) | Never | 69.81(1,683) | 71.63(1,381) | 75.10(380) | 0.007 |
|  | Less than once a month | 5.68(137) | 6.74(130) | 7.11(36) |  |
|  | More than once a month | 24.51(591) | 21.63(417) | 17.79(90) |  |
| Social activities engagement, %(n) | Yes | 53.96(1,301) | 50.16(967) | 42.09(213) | < 0.001 |
|  | No | 46.04(1,110) | 49.84(961) | 57.91(293) |  |
| Sleep duration at night, hours, %(n) | 0 ~ 4 | 5.18(125) | 12.81(247) | 23.12(117) | < 0.001 |
|  | 4 ~ 6 | 18.75(452) | 29.51(569) | 35.38(179) |  |
|  | 6 ~ 8 | 45.91(1,107) | 33.77(651) | 24.11(122) |  |
|  | ≥ 8 | 30.15(727) | 23.91(461) | 17.39(88) |  |
| *^*^*BMI degree, %(n) | Underweight | 4.73(114) | 7.99(154) | 9.49(48) | < 0.001 |
|  | Normal weight | 38.53(929) | 39.37(759) | 36.56(185) |  |
|  | Overweight | 17.92(432) | 17.69(341) | 17.39(88) |  |
|  | Obesity | 38.82(936) | 34.96(674) | 36.56(185) |  |
| Co-morbidities, %(n) | Yes | 56.12(1,353) | 69.29(1,336) | 81.23(411) | < 0.001 |
|  | No | 43.88(1,058) | 30.71(592) | 18.77(95) |  |
| Hypertension, %(n) |  | 45.25(1,091) | 49.53(955) | 46.84(237) | 0.019 |
| Abdominal adiposity, %(n) |  | 45.21(1,090) | 44.40(856) | 45.85(232) | 0.791 |
| Diabetes, %(n) |  | 11.57(279) | 15.30(295) | 17.59(89) | < 0.001 |
| Dyslipidemia, %(n) |  | 42.34(1,020) | 40.25(776) | 39.09(197) | < 0.001 |
| *^*^*CKD, %(n) |  | 57.49(1,386) | 62.60(1,207) | 63.83(323) | < 0.001 |
| Cancer, %(n) |  | 0.95(23) | 1.19(23) | 1.19(6) | 0.725 |
| Chronic pain, %(n) |  | 15.72(379) | 40.20(775) | 63.64(322) | < 0.001 |
| *^*^*CRP (mg/L), mean (SD) |  | 2.75(7.06) | 2.87(7.75) | 2.93(6.70) | < 0.001 |
| *^*^*MCV, mean (SD) |  | 90.55(8.62) | 90.47(8.64) | 90.85(9.21) | 0.220 |
| *^*^*CES-D-10 scores, mean (SD) |  | 4.64(2.75) | 13.60(2.75) | 22.88(2.51) | < 0.001 |
| Physical symptoms scores |  | 2.37(2.00) | 7.10(2.20) | 11.87(1.84) | < 0.001 |
| Depressive emotion scores |  | 0.77(1.07) | 3.30(1.68) | 6.31(1.79) | < 0.001 |
| Positive mood scores |  | 1.49(1.66) | 3.20(1.70) | 4.69(1.43) | < 0.001 |
| *^*^Variables are presented as percentages (number), or mean (SD).* | | | | | |
| *^*^Abbreviation: PSM, Propensity score matching; BMI, body mass index; CKD, Chronic kidney disease; CRP, C-reactive protein; MCV, Mean Corpuscular Volume; NDS, non-depressive symptom; DS, depressive symptom; DD, depressive disorder; CES-D-10, 10-item short form of the Center for Epidemiologic Studies Depression Scale.* | | | | | |
| *^*^P-value less than 0.05 was defined as significant.* | | | | |  |
