# Supplementary material for: Relationship between depressive symptoms and anemia among the middle-aged and elderly: a cohort study over 4-year period
Source: BMC Psychiatry. 2023 Aug 8;23:572. doi: 10.1186/s12888-023-05047-6 (PMC10408197; doi:10.1186/s12888-023-05047-6)
Supplement: Supplementary file 4 — Additional file 4: Supplement Table 4. Characteristics of participants after PSM for a 1:2 matched design in longitudinal study (2015, N = 2,750). [file 12888_2023_5047_MOESM4_ESM.docx]

| **Supplement Table 4 Characteristics of participants after PSM for a 1:2 matched design in longitudinal study (2015, N = 2,750)** | | | | | |
| --- | --- | --- | --- | --- | --- |
| Variables | Assignment description | Depressive Symptoms | | |  |
|  |  | NDS group N=1,381 (50.22%) | DS group N=1,098 (39.93%) | DD group N=271 (9.85%) | P-value |
| Anemia, %(n) |  | 12.31(170) | 17.30(190) | 20.66(56) | < 0.001 |
| Hemoglobin (g/dL), mean (*^*^*SD) |  | 13.92(1.79) | 13.69(1.90) | 13.61(1.91) | 0.018 |
| Age, year, mean (SD) |  | 58.08(8.72) | 59.81(9.53) | 60.66(8.96) | 0.028 |
| Age, year, %(n) | 45-59 | 60.03(829) | 53.10(583) | 50.55(131) | < 0.001 |
|  | ≥60 | 39.97(552) | 46.90(515) | 49.45(134) |  |
| Gender, %(n) | Male | 52.79(729) | 57.38(630) | 64.21(174) | 0.001 |
|  | Female | 47.21(652) | 42.62(468) | 35.79(97) |  |
| Educational level, %(n) | Illiterate | 21.00(290) | 29.69(326) | 42.44(115) | < 0.001 |
|  | Primary education | 43.52(601) | 47.09(517) | 41.70(113) |  |
|  | Secondary education | 34.11(471) | 22.68(249) | 15.50(42) |  |
|  | Higher education | 1.38(19) | 0.55(6) | 0.37(1) |  |
|  | Postgraduate education | 0(0) | 0(0) | 0(0) |  |
| Marital status, %(n) | Single | 0.51(7) | 1.55(17) | 1.48(4) |  |
|  | Married | 87.55(1,209) | 80.33(882) | 73.06(198) |  |
|  | Divorced | 0.43(6) | 1.28(14) | 2.21(6) |  |
|  | Widowed | 11.51(159) | 16.85(185) | 23.25(63) |  |
| Residence, %(n) | Rural | 86.82(1,199) | 87.25(958) | 94.10(255) | 0.003 |
|  | Urban | 13.18(182) | 12.75(140) | 5.90(16) |  |
| Smoking status, %(n) | Never | 59.81(826) | 60.47(664) | 63.47(172) | 0.032 |
|  | Quit | 7.75(107) | 8.74(96) | 12.18(33) |  |
|  | Current | 32.44(448) | 30.78(338) | 24.35(66) |  |
| Alcohol consumption, %(n) | Never | 67.41(931) | 69.31(761) | 75.65(205) | 0.035 |
|  | Less than a month | 6.81(94) | 7.74(85) | 7.01(19) |  |
|  | More than a month | 25.78(356) | 22.95(252) | 17.34(47) |  |
| Social activities engagement, %(n) | Yes | 54.74(756) | 50.91(559) | 40.96(111) | < 0.001 |
|  | No | 45.26(625) | 49.09(539) | 59.04(160) |  |
| Sleep duration at night, hours, %(n) | 0 ~ 4 | 4.56(63) | 12.20(134) | 22.88(62) | < 0.001 |
|  | 4 ~ 6 | 19.84(274) | 29.69(326) | 33.21(90) |  |
|  | 6 ~ 8 | 45.11(623) | 34.70(381) | 26.57(72) |  |
|  | ≥ 8 | 30.49(421) | 23.41(257) | 17.34(47) |  |
| *^*^*BMI degree, %(n) | Underweight | 3.69(51) | 6.74(74) | 6.27(17) | 0.011 |
|  | Normal weight | 36.13(499) | 38.80(426) | 36.90(100) |  |
|  | Overweight | 20.20(279) | 18.67(205) | 19.19(52) |  |
|  | Obesity | 39.97(552) | 35.79(393) | 37.64(102) |  |
| Co-morbidities, %(n) | Yes | 58.36(806) | 68.12(748) | 80.07(217) | < 0.001 |
|  | No | 41.64(575) | 31.88(350) | 19.93(54) |  |
| Hypertension, %(n) |  | 44.10(609) | 50.55(555) | 52.03(141) | 0.002 |
| Abdominal adiposity, %(n) |  | 49.17(679) | 46.72(513) | 50.18(136) | 0.387 |
| Diabetes, %(n) |  | 12.60(174) | 13.21(145) | 17.34(47) | 0.019 |
| Dyslipidemia, %(n) |  | 46.34(640) | 39.53(434) | 46.13(125) | 0.002 |
| *^*^*CKD, %(n) |  | 55.61(768) | 60.93(669) | 60.89(165) | 0.019 |
| Cancer, %(n) |  | 0.80(11) | 1.28(14) | 1.85(5) | 0.237 |
| Chronic pain, %(n) |  | 16.22(224) | 39.80(437) | 68.27(185) | < 0.001 |
| *^*^*CRP (mg/L), mean (SD) |  | 2.57(6.61) | 2.58(5.34) | 2.81(4.93) | < 0.001 |
| *^*^*MCV, mean (SD) |  | 91.32(7.38) | 91.08(8.14) | 91.73(8.67) | 0.306 |
| *^*^*CES-D-10 scores, mean (SD) |  | 4.71(2.74) | 13.62(2.75) | 22.82(2.36) | < 0.001 |
| Physical symptoms scores |  | 2.37(1.99) | 7.11(2.18) | 11.94(1.78) | < 0.001 |
| Depressive emotion scores |  | 1.54(1.69) | 3.22(1.70) | 4.68(1.38) | < 0.001 |
| Positive mood scores |  | 0.80(1.08) | 3.29(1.63) | 6.20(1.70) | < 0.001 |
| *^*^Variables are presented as percentages (number), or mean (SD).* | | | | | |
| *^*^Abbreviation: PSM, Propensity score matching; BMI, body mass index; CKD, Chronic kidney disease; CRP, C-reactive protein; MCV, Mean Corpuscular Volume; NDS, non-depressive symptom; DS, depressive symptom; DD, depressive disorder; CES-D-10, 10-item short form of the Center for Epidemiologic Studies Depression Scale.* | | | | | |
| *^*^P-value less than 0.05 was defined as significant.* | | | | | |
