# Supplementary material for: Relationship between depressive symptoms and anemia among the middle-aged and elderly: a cohort study over 4-year period
Source: BMC Psychiatry. 2023 Aug 8;23:572. doi: 10.1186/s12888-023-05047-6 (PMC10408197; doi:10.1186/s12888-023-05047-6)
Supplement: Supplementary file 5 — Additional file 5: Supplement Table 5. Gender subgroup analysis: the relationship between different depressive symptoms group, scores and anemia in cross-sectional study (2011). [file 12888_2023_5047_MOESM5_ESM.docx]

| **Supplement Table 5 Gender subgroup analysis: The relationship between different depressive symptoms group, scores and anemia in cross-sectional study (2011)** | | | | | | | | |
| --- | --- | --- | --- | --- | --- | --- | --- | --- |
|  | **Male** | | | | | | | |
|  | Model 1^a^ | |  | Model 2^b^ | |  | Model 3^c^ | |
|  | OR (95% CI) | P |  | OR (95% CI) | P |  | OR (95% CI) | P |
| NDS group (N= 3,296) | 1(reference) |  |  | 1(reference) |  |  | 1(reference) |  |
| DS group (N=1,248) | 1.14(0.88-1.47) | 0.321 |  | 1.12(0.86-1.46) | 0.394 |  | 1.08(0.83-1.41) | 0.571 |
| DD group (N=250) | 1.80(1.18-2.74) | 0.006 |  | 1.71(1.11-2.63) | 0.014 |  | 1.72(1.11-2.65) | 0.014 |
|  |  |  |  |  |  |  |  |  |
| CES-D-10 scores | 1.03(1.01-1.04) | 0.001 |  | 1.02(1.00-1.04) | 0.05 |  | 1.01(0.99-1.03) | 0.335 |
| Physical symptoms scores | 1.04(1.01-1.06) | 0.006 |  | 1.04(1.01-1.06) | 0.012 |  | 1.03(1.00-1.06) | 0.024 |
| Depressed emotion scores | 1.06(1.00-1.11) | 0.047 |  | 1.05(0.99-1.11) | 0.083 |  | 1.05(0.99-1.11) | 0.082 |
| Optimistic mood scores | 1.09(1.04-1.14) | <0.001 |  | 1.05(1.00-1.10) | 0.031 |  | 1.07(1.02-1.12) | 0.005 |
|  | **Female** | | | | | | | |
|  | Model 1^a^ | |  | Model 2^b^ | |  | Model 3^c^ | |
|  | OR (95% CI) | P |  | OR (95% CI) | P |  | OR (95% CI) | P |
| NDS group (N= 2,996) | 1(reference) |  |  | 1(reference) |  |  | 1(reference) |  |
| DS group (N= 1,882) | 1.02(0.81-1.27) | 0.891 |  | 1.04(0.82-1.31) | 0.748 |  | 1.03(0.81-1.30) | 0.808 |
| DD group (N= 507) | 1.22(0.87-1.70) | 0.235 |  | 1.25(0.89-1.77) | 0.199 |  | 1.20(0.84-1.70) | 0.325 |
|  |  |  |  |  |  |  |  |  |
| CES-D-10 scores | 1.00(0.99-1.10) | 0.889 |  | 1.00(0.99-1.02) | 0.405 |  | 1.00(0.98-1.02) | 0.646 |
| Physical symptoms scores | 1.00(0.98-1.02) | 0.814 |  | 1.00(0.98-1.03) | 0.675 |  | 1.00(0.98-1.02) | 0.933 |
| Depressed emotion scores | 1.03(0.98-1.07) | 0.264 |  | 1.03(0.98-1.08) | 0.197 |  | 1.02(0.98-1.07) | 0.309 |
| Optimistic mood scores | 1.00(0.97-1.05) | 0.808 |  | 1.00(0.96-1.04) | 0.866 |  | 1.00(0.96-1.04) | 0.901 |
| ^a^Adjusted for demographic variables (including age, gender, education, marital status, residence). | | | | | |  |  |  |
| ^b^Adjusted for demographic and behavioral variables (including smoking status, alcohol consumption, social participation and daily sleep duration) | | | | | | | | |
| ^c^Adjusted for demographic, behavioral and disease-related variables (including BMI, CRP, hypertension, diabetes, dyslipidemia, abdominal obesity, chronic lung disease, heart disease, stroke, cancer, chronic kidney disease, hepatopathy, asthma and chronic pain) | | | | | | | | |
| ^*^Abbreviation: OR, odds ratio; CI confidence intervals; NDS, non-depressive symptom; DS, depressive symptom; DD, depressive disorder; CES-D-10, Center for Epidemiologic Studies Depression Scale. | | | | | | | | |
