# Supplementary material for: Relationship between depressive symptoms and anemia among the middle-aged and elderly: a cohort study over 4-year period
Source: BMC Psychiatry. 2023 Aug 8;23:572. doi: 10.1186/s12888-023-05047-6 (PMC10408197; doi:10.1186/s12888-023-05047-6)
Supplement: Supplementary file 6 — Additional file 6: Supplement Table 6. Gender subgroup analysis: longitudinal association between different depressive symptoms groups, scores and anemia (2015). [file 12888_2023_5047_MOESM6_ESM.docx]

| **Supplement Table 6 Gender subgroup analysis: Longitudinal association between different depressive symptoms groups, scores and anemia (2015)** | | | | | | | | |
| --- | --- | --- | --- | --- | --- | --- | --- | --- |
|  | **Male** | | | | | | | |
|  | Model 1^a^ | |  | Model 2^b^ | |  | Model 3^c^ | |
|  | OR (95% CI) | P |  | OR (95% CI) | P |  | OR (95% CI) | P |
| NDS group (N= 1,942) | 1(reference) |  |  | 1(reference) |  |  | 1(reference) |  |
| DS group (N=691) | 1.02(0.78-1.33) | 0.873 |  | 0.98(0.75-1.29) | 0.926 |  | 0.89(0.628-1.25) | 0.494 |
| DD group (N=134) | 2.14(1.38-3.33) | 0.001 |  | 2.02(1.29-3.15) | 0.002 |  | 2.19(1.27-3.75) | 0.005 |
|  |  |  |  |  |  |  |  |  |
| CES-D-10 scores | 1.03(1.01-1.05) | 0.01 |  | 1.03(1.00-1.05) | 0.037 |  | 1.02(1.00-1.04) | 0.047 |
| Physical symptoms scores | 1.34(1.00-1.07) | 0.004 |  | 1.03(1.00-1.07) | 0.05 |  | 1.02(0.99-1.06) | 0.239 |
| Depressed emotion scores | 1.13(1.05-1.21) | 0.001 |  | 1.12(1.04-1.21) | 0.002 |  | 1.10(1.03-1.19) | 0.007 |
| Optimistic mood scores | 1.06(1.00-1.12) | 0.042 |  | 1.04(0.99-1.11) | 0.174 |  | 1.02(0.98-1.07) | 0.315 |
|  | **Female** | | | | | | | |
|  | Model 1^a^ | |  | Model 2^b^ | |  | Model 3^c^ | |
|  | OR (95% CI) | P |  | OR (95% CI) | P |  | OR (95% CI) | P |
| NDS group (N= 1,725) | 1(reference) |  |  | 1(reference) |  |  | 1(reference) |  |
| DS group (N=1,100) | 1.40(1.01- 1.94) | 0.04 |  | 1.40(1.01-1.56) | 0.043 |  | 1.42(0 .89-2.25) | 0.142 |
| DD group (N=295) | 1.45(1.19-1.78) | <0.001 |  | 1.45(1.18-1.78) | <0.001 |  | 1.54(1.16-2.06) | 0.003 |
|  |  |  |  |  |  |  |  |  |
| CES-D-10 scores | 1.03 (1.01-1.05) | 0.001 |  | 1.03(1.01-1.05) | 0.001 |  | 1.02(1.01-1.04) | 0.005 |
| Physical symptoms scores | 1.04(1.02-1.07) | <0.001 |  | 1.05(1.02-1.07) | <0.001 |  | 1.04(1.02-1.07) | 0.002 |
| Depressed emotion scores | 1.12(1.05-1.19) | 0.001 |  | 1.12(1.04-1.19) | 0.001 |  | 1.11(1.04-1.19) | 0.002 |
| Optimistic mood scores | 1.04(1.00-1.09) | 0.05 |  | 1.04(0.99-1.08) | 0.104 |  | 1.03(0.97-1.10) | 0.286 |
| ^a^Adjusted for demographic variables (including age, gender, education, marital status, residence). | | | | | |  |  |  |
| ^b^Adjusted for demographic and behavioral variables (including smoking status, alcohol consumption, social participation and daily sleep duration) | | | | | | | | |
| ^c^Adjusted for demographic, behavioral and disease-related variables (including BMI, CRP, hypertension, diabetes, dyslipidemia, abdominal obesity, chronic lung disease, heart disease, stroke, cancer, chronic kidney disease, hepatopathy, asthma and chronic pain) | | | | | | | | |
| ^*^Abbreviation: OR, odds ratio; CI confidence intervals; NDS, non-depressive symptom; DS, depressive symptom; DD, depressive disorder; CES-D-10, Center for Epidemiologic Studies Depression Scale. | | | | | | | | |
