# Supplementary material for: Relationship between depressive symptoms and anemia among the middle-aged and elderly: a cohort study over 4-year period
Source: BMC Psychiatry. 2023 Aug 8;23:572. doi: 10.1186/s12888-023-05047-6 (PMC10408197; doi:10.1186/s12888-023-05047-6)
Supplement: Supplementary file 7 — Additional file 7: Supplement Table 7. Age subgroup analysis: the relationship between different depressive symptoms group, scores and anemia in cross-sectional study (2011). [file 12888_2023_5047_MOESM7_ESM.docx]

| **Supplement Table 7 Age subgroup analysis: The relationship between different depressive symptoms group, scores and anemia in cross-sectional study (2011)** | | | | | | | | |
| --- | --- | --- | --- | --- | --- | --- | --- | --- |
|  | **Age < 60** | | | | | | | |
|  | Model 1^a^ | |  | Model 2^b^ | |  | Model 3^c^ | |
|  | OR (95% CI) | P |  | OR (95% CI) | P |  | OR (95% CI) | P |
| NDS group (N=3,653) | 1(reference) |  |  | 1(reference) |  |  | 1(reference) |  |
| DS group (N=1,595) | 0.97(0.75-1.25) | 0.814 |  | 1.00(0.77-1.30) | 0.990 |  | 0.98(0.76- 1.28） | 0.905 |
| DD group (N=359) | 1.34(0.90-1.98) | 0.149 |  | 1.39(0.92-2.08) | 0.116 |  | 1.36(.90-2.06) | 0.145 |
|  |  |  |  |  |  |  |  |  |
| CES-D-10 scores | 1.01(0.99-1.02) | 0.545 |  | 1.01(0.99-1.03) | 0.388 |  | 1.01(0.98-1.02) | 0.573 |
| Physical symptoms scores | 1.02(1.00-1.04) | 0.113 |  | 1.01(0.99-1.04) | 0.303 |  | 1.01(0.98-1.03) | 0.682 |
| Depressed emotion scores | 1.01(0.96-1.07) | 0.654 |  | 1.02(0.96-1.07) | 0.527 |  | 1.01(0.96-1.07) | 0.604 |
| Optimistic mood scores | 1.03(0.99-1.08) | 0.17 |  | 1.03(0.98-1.08) | 0.216 |  | 1.03(0.99-1.08) | 0.175 |
|  | **Age>=60** | | | | | | | |
|  | Model 1^a^ | |  | Model 2^b^ | |  | Model 3^c^ | |
|  | OR (95% CI) | P |  | OR (95% CI) | P |  | OR (95% CI) | P |
| NDS group (N=2,639) | 1(reference) |  |  | 1(reference) |  |  | 1(reference) |  |
| DS group (N=1,535) | 1.16(0.93-1.46) | 0.192 |  | 1.16(0.92-1.47) | 0.212 |  | 1.13(0 .89-1.43) | 0.328 |
| DD group (N= 398) | 1.49(1.05-2.12) | 0.024 |  | 1.45(1.02-2.08) | 0.041 |  | 1.40(0.97-2.02) | 0.073 |
|  |  |  |  |  |  |  |  |  |
| CES-D-10 scores | 1.01(1.00-1.03) | 0.025 |  | 1.01 (1.00-1.03) | 0.075 |  | 1.01(0.99-1.03) | 0.379 |
| Physical symptoms scores | 1.02(1.00-1.05) | 0.032 |  | 1.02 (1.00-1.05) | 0.038 |  | 1.02(1.00-1.05) | 0.063 |
| Depressed emotion scores | 1.06(1.01-1.11) | 0.016 |  | 1.05(1.01-1.11) | 0.026 |  | 1.05(1.01-1.10) | 0.046 |
| Optimistic mood scores | 1.03(0.99-1.07) | 0.188 |  | 1.01(0.96-1.05) | 0.804 |  | 1.02(0.98-1.06) | 0.427 |
| ^a^Adjusted for demographic variables (including age, gender, education, marital status, residence). | | | | | |  |  |  |
| ^b^Adjusted for demographic and behavioral variables (including smoking status, alcohol consumption, social participation and daily sleep duration) | | | | | | | | |
| ^c^Adjusted for demographic, behavioral and disease-related variables (including BMI, CRP, hypertension, diabetes, dyslipidemia, abdominal obesity, chronic lung disease, heart disease, stroke, cancer, chronic kidney disease, hepatopathy, asthma and chronic pain) | | | | | | | | |
| ^*^Abbreviation: OR, odds ratio; CI confidence intervals; NDS, non-depressive symptom; DS, depressive symptom; DD, depressive disorder; CES-D-10, Center for Epidemiologic Studies Depression Scale. | | | | | | | | |
