# Supplementary material for: Relationship between depressive symptoms and anemia among the middle-aged and elderly: a cohort study over 4-year period
Source: BMC Psychiatry. 2023 Aug 8;23:572. doi: 10.1186/s12888-023-05047-6 (PMC10408197; doi:10.1186/s12888-023-05047-6)
Supplement: Supplementary file 8 — Additional file 8: Supplement Table 8. Age subgroup analysis: longitudinal association between different depressive symptoms groups, scores and anemia (2015). [file 12888_2023_5047_MOESM8_ESM.docx]

| **Supplement Table 8 Age subgroup analysis: Longitudinal association between different depressive symptoms groups, scores and anemia (2015)** | | | | | | | | |
| --- | --- | --- | --- | --- | --- | --- | --- | --- |
|  | **Age < 60** | | | | | | | |
|  | Model 1^a^ | |  | Model 2^b^ | |  | Model 3^c^ | |
|  | OR (95% CI) | P |  | OR (95% CI) | P |  | OR (95% CI) | P |
| NDS group (N=3,653) | 1(reference) |  |  | 1(reference) |  |  | 1(reference) |  |
| DS group (N=1,595) | 1.74(1.29-2.37) | <0.001 |  | 1.73(1.26-2.37) | 0.001 |  | 1.65(1.20-2.27) | 0.002 |
| DD group (N=359) | 2.02(1.24-3.28) | 0.005 |  | 1.93(1.16-3.22) | 0.011 |  | 1.89(1.12-3.18) | 0.017 |
|  |  |  |  |  |  |  |  |  |
| CES-D-10 scores | 1.05(1.02-1.07) | <0.001 |  | 1.05(1.02-1.07) | <0.001 |  | 1.04(1.02-1.07) | <0.001 |
| Physical symptoms scores | 1.08(1.04-1.12) | <0.001 |  | 1.08(1.03-1.12) | <0.001 |  | 1.07(1.03-1.12) | 0.001 |
| Depressed emotion scores | 1.05(1.00-1.10) | 0.057 |  | 1.04(0.99-1.10) | 0.096 |  | 1.03(0.98-1.09) | 0.232 |
| Optimistic mood scores | 1.18(1.10-1.27) | <0.001 |  | 1.17(1.09-1.26) | <0.001 |  | 1.16(1.08-1.25) | <0.001 |
|  | **Age>=60** | | | | | | | |
|  | Model 1^a^ | |  | Model 2^b^ | |  | Model 3^c^ | |
|  | OR (95% CI) | P |  | OR (95% CI) | P |  | OR (95% CI) | P |
| NDS group (N=2,639) | 1(reference) |  |  | 1(reference) |  |  | 1(reference) |  |
| DS group (N=1,535) | 1.08(0.81-1.43) | 0.610 |  | 1.66(0.79-1.42) | 0.676 |  | 1.00 (0.74-1.35) | 0.997 |
| DD group (N= 398) | 1.44(0.92-2.25) | 0.111 |  | 1.43(0.90-2.28) | 0.130 |  | 1.32(0.82-2.12) | 0.259 |
|  |  |  |  |  |  |  |  |  |
| CES-D-10 scores | 1.02(1.00-1.04) | 0.091 |  | 1.02(1.00-1.04) | 0.115 |  | 1.01(0.99-1.03) | 0.325 |
| Physical symptoms scores | 1.03(0.99-1.06) | 0.123 |  | 1.03(0.99-1.07) | 0.161 |  | 1.02(0.98-1.06) | 0.367 |
| Depressed emotion scores | 1.03(0.99-1.09) | 0.171 |  | 1.03(0.98-1.08) | 0.218 |  | 1.02(0.97-1.07) | 0.446 |
| Optimistic mood scores | 1.08(1.01-1.15) | 0.021 |  | 1.08(1.01-1.15) | 0.022 |  | 1.06(0.99-1.14) | 0.077 |
| ^a^Adjusted for demographic variables (including age, gender, education, marital status, residence). | | | | | |  |  |  |
| ^b^Adjusted for demographic and behavioral variables (including smoking status, alcohol consumption, social participation and daily sleep duration) | | | | | | | | |
| ^c^Adjusted for demographic, behavioral and disease-related variables(including BMI, CRP, hypertension, diabetes, dyslipidemia, abdominal obesity, chronic lung disease, heart disease, stroke, cancer, chronic kidney disease, hepatopathy, asthma and chronic pain) | | | | | | | | |
| ^*^Abbreviation: OR, odds ratio; CI confidence intervals; NDS, non-depressive symptom; DS, depressive symptom; DD, depressive disorder; CES-D-10, Center for Epidemiologic Studies Depression Scale. | | | | | | | | |
